# The Siamese Twins—Autopsy, Etc.

**Published:** 1874-03

**Authors:** 


*PHILADELPHIA MEDICAL TIMES—February*, 1874:

### PHONOGRAPHIC REPORT.

### THE SIAMESE TWINS AT THE COLLEGE OF PHYSICIANS.

A special meeting of the College of Physicians of Philadelphia was held at the hall, Wednesday evening, February 18, for the purpose of hearing the report of the Commission on the Siamese Twins,—Dr. W. S. W. Ruschenberger, U. S. N., in the chair. On motion of Dr. Gross, it was, after some discussion, resolved that the Philadelphia Medical Times, be allowed to report the proceedings of the meeting, on condition that three hundred copies of the journal should be given to the college for the use of the members.

The bodies of the Siamese Twins being upon the table, the meeting proceeded to hear the report of Drs. Pancoast and Allen. On behalf of the Commission, Dr. Pancoast stated that, the dissection not having been entirely completed, their report would be a verbal one, to be followed at some later date by a memoir upon the subject. He further remarked that it had been agreed that he should consider chiefly the surgical aspect of the matter in hand, whilst to his colleague had been assigned the demonstration of the anatomical peculiarities.

Dr. William H. Pancoast:

Mr. Chairman, and Fellows of the College:—Having been requested, as a member of the Commission, to open the discussion this evening, I will say briefly, in reference to this monster of a symmetrical duplex development, joined, as many of the Fellows, now know, at the ensiform appendix and also here at the omphalos or navel, that at the investigation which we made on the first occasion at Mount Airy I made the opening incision of the body on the line for the ligation of the primitive iliac, on the right side; Dr. Allen made the incision on the left. The object was to reach the great vessels,—the aorta and two primitive iliacs, and to force the injecting material which we used for embalming (chloride of zinc) up the aorta and down the iliacs until it ran from the incisions made in the fingers and toes. It flowed freely through the bloodvessels in Eng, owing to the ossified condition of his arteries; the injection in Chang was, however, not so successful, owing to decomposition in the tissues and blood-vessels. It was necessary to repeat the injecting process several times in order to preserve the body. The arteries of Chang were found to be very much decomposed,—quite rotten, in fact.

In Dunglison's Medical Dictionary we find the scientific name given for the Siamese Twins, in the classification of teratology, to be *Xiphopages;* and by referring to the admirable article on Diploteratology of Dr. G. J. Fisher, (published in the Transactions of the Medical Society of the State of New York for the year 1866), it will be found that the twins belong in the class of *Anacatadidyma.* In his classification of double monsters he makes three orders: *Order first,—Teratacatadidyma;* derived from *τέρας, τέρατος*, a "monster," *κατά*, "down," and *δίδυμος*, a "twin." *Definition,*—duplicity, with more or less separation, of the cerebro-spinal axis, from above downwards. *Order second,—Terata-anadidyma,* derived from *ἀνά*, "up" or "above," and *δίδυμος*, a "twin." *Definition,*—duplicity, with more or less separation, of the cerebro-spinal axis, from below upwards, or from the caudal towards the cephalic extremity of the neural axis. *Order third,—Terata-anacatadidyma,* derived from *ἀνά*, "above," *κατά*, "down," and *δίδυμος*, a "twin." *Definition,*—duplicity, with more or less separation, of both the cephalic and the caudal extremity of the cerebro-spinal axis, existing contemporaneously. In this order, the monster now before us might be called an *Omphelopagus Xiphodidymus.*

Thus we have the scientific nomenclature of this monster. Of course, the consideration of greatest interest to the profession, and one of the main reasons why the Commission made such exertions to obtain this post-mortem, was that the American profession might not be charged for having neglected an effort to obtain an autopsy, which would solve the mystery of their union. The feature of greatest interest is connected with this band,—about four inches long and eight inches in circumference. In addition to this, there are other points of importance in teratology, in regard to the fulfilment of the law of homologous union, in relation to the juncture of the recti muscles and the fasciæ of the obliqus and transversalis at their point of meeting in the centre of the band. In regard to the position of the hearts, we think their apices present towards each other; but we have not yet opened the thorax. The livers we have found to approximate to each other and to push through the respective peritoneal openings into the band. We extended our incisions to the margin of the band in front. By placing my hand in the peritoneal cavity of Eng, and my colleague placing his hand in the peritoneal cavity of Chang, we pushed before us processes of peritoneum, which ran on to the median line of the band; and we could feel our fingers in the lower portion of the band, behind the median line, with a distinct layer of peritoneum between them, demonstrating at once the prolongation of the peritoneum into the band, and the complete separation of one peritoneal cavity from the other at this median line. Above that we felt some traces of vascular connection, apparently running from one liver to the other; but this we will examine into when we have a better opportunity of carefully dissecting and examining what vascular structures may exist. We also noticed that in turning off the flaps consisting of the anterior walls of the abdomen, the hypogastric arteries, as illustrated by the diagram on the blackboard, ran upwards in each body into the band. We lost them in this way, as we think, towards the common umbilicus in the anterior inferior surface of the middle of the band.

It is probable that the two hypogastric arteries on each side passed through this umbilicus. Whether or not there were two umbilical veins, we have not yet been able to decide, nor to answer the question whether the umbilical cord was double or single and composed of the four hypogastric arteries and two umbilical veins, or whether the placenta was single, double, or twin.

We also recognized that the ensiform appendix, as shown in the diagram of each side, was prolonged and united in the middle line. On our later examination, we find that there is complete continuity of structure of the cartilages, but no true joint at the middle line, although it is possible there may be some small synovial sacs farther up. The motion is mainly due, as I here demonstrate to you by moving these bodies one upon the other, to the elasticity of the connected ensiform appendices and intervening fibro-cartilages.

In regard to the vascular connection of the band, we have not yet been able to make so thorough and careful an examination as we wished; but still, in throwing colored plaster into the portal circulation of Chang it has been found to flow through the vessels of the upper part of the band into the portal vessels of Eng. So that the surgical anatomy of the band consists in the skin and fascia which cover it, the two separate peritoneal pouches which meet in the middle, the large peritoneal pouch, the vascular connection, to whatever extent that may exist between the two portal circulations, and the remains of the hypogastric arteries in the lower portion of the band. Thus the main difficulty in any operation for section of the band would seem to be in regard to the peritoneal processes and the portal circulation. The anastomosis which may exist between the internal mammary arteries and the intercostals in the integument in the upper portion of the band, of course would present no difficulty.

I will not venture upon any further remarks as to the surgery of the case, while there are so many distinguished gentlemen present more competent than myself to give an opinion. At the same time, operations on the peritoneum may not be considered so hazardous in this day, when ovariotomy, gastrotomy, and even Cæsarian section, are so often performed. The peritoneum-pouches themselves would not present so great a difficulty as might be anticipated, under pressure and acupuncture, by which the sensitiveness of the structure might be so altered as to permit of a section. I was informed at Mount Airy that in Paris a surgeon had made the experiment of applying pressure upon the band, and it was reported the twins had fainted in consequence. I could not ascertain, however, whether this was from fright, design, or actual pain.

As Dr. Hollingsworth is present, it may be proper for me to mention a fact which that gentleman can corroborate, that Eng was the stronger physically and Chang was the stronger mentally. The same difference was observable in their characters. Chang was more irritable than Eng, especially since an attack of paralysis with which he had been afflicted,—this being in the side next to Eng. The latter had not only to bear with the irritability of his associate, but also to support one-half his weight. Among other peculiarities, Chang would sometimes break useful articles or throw them in the fire.

In conclusion, let me say that when I turned up the skin and superficial fascia of the H incision on the posterior part of the band, I was struck with the development and the strength of the abdominal aponeuroses. The fibres arched, interlaced, and developed into a strong fibrous band about a quarter of an inch wide, running around the median line, although there was no actual joint in the cartilage.

Prof. HARRISON ALLEN:

Mr. Chairman:—I will probably best discharge the duty devolving upon me by at once proceeding to a somewhat more minute anatomical description than Dr. Pancoast has given, this being in accordance with the understanding between us in reference to the evening's exercises.

Perhaps it would be best to point to that simple diagram upon the blackboard before considering the subject more fully in detail. As Dr. Pancoast has informed the Fellows, there is a union of the twins at the two ensiform cartilages, which are very firmly joined in the centre, Eng's process being the more robust of the two. You will observe that there is a point of conjunction between the two processes which is not quite in the median line of the band. In the centre of the band is seen an eliptical space which suggests to the observer the presence of a synovial cavity. It is probable that the ensiform junction is of the character of a synchondrosis, with a median bursa-like sac; neither ensiform cartilage is ossified.

Below this point, in the diagram, you see a number of differently-lined tracks. The lower one, immediately above the umbilicus, is only separated from the skin by a very delicate layer of tissue (so that, with the finger introduced into the pouch and moved, there is a decided indication of motion in the skin) on the under surface of the band.

This pouch passes across the band from the abdomen of Chang, and is lost in the duplicature of the suspensory ligament of the liver of Eng. The finger passed upward to the band from the abdomen of Eng crosses the band above the pouch just mentioned, and is lost between the layers of the suspensory ligament of the liver of Chang. When the significance of the round ligament at the free border of the suspensory ligament is remembered, the relations of these pouches directly suggest that they have had essential bearings to the umbilical vein of the funis, and may be provisionally termed the *umbilical pouches.*

Above Eng's pouch, and between it and the under surface of the ensiform conjunction, is a second pouch prolonged from Chang's abdomen, which fairly reaches the peritoneal cavity of Eng, but is not continuous with it. Extending up into this pouch from Chang's abdomen is a process which suggested to the Commission the possibility of the transit of hepatic vessels. This view was rendered more probable from the fact that a similar process passed up into the band from the liver of Eng. Accordingly, the plaster injection, colored by ultra-marine, was thrown into a tributary of the portal vein of Chang, when it was observed that the fluid passed freely into the liver of Eng, as well as into some of the mesenteric veins proper. It is my own hypothesis that this bond of union was the true hepatic tract; but in its present state, in the absence of evidence of any parenchymatous admixture about the vessels thus crossing the band, we prefer to denominate the transit as the *tract of portal continuity.*

In the fœtal condition it is very likely that this large space, the upper pouch, now continuous with the abdomen of Chang only, was entirely occupied by true liver-tissue, which, as mturity was attained, became smaller, and left an empty space. Hence I propose to call this upper pouch the *hepatic pouch.* The contraction chanced to be greater on Chang's side, in harmony, it may be, with other evidences of a weaker and less developed type, which is so apparent in many of the tissues of Chang. Now, with reference to the demonstration. As Dr. Pancoast has already informed you, the incisions in the abdomen were made in rather an exceptional manner. By reference to the parts it will be seen that the incision in either individual was located in such a way as to avoid the median line, since it was supposed from the peculiar position of the umbillicus that the remains of the hypogastric arteries would be found extending from the fundus of the bladder upward and inward along the entire length of the anterior wall of the abdomen. Besides, this incision would enable us, by continuing from below upward, to fairly open the abdomen and examine the cord, without violating the conditions by which the Commission was bound. The flap comprises the greater part of the abdominal wall, and can be best observed, from the position of the bodies on the table, in that of Eng.

You notice that the tissues are well supplied with fat; and this condition is very plainly in contrast with that of Chang. Eng's side of the band is well nourished; Chang's end of the band presents an entirely different aspect. Chang was an invalid, and the weaker half of this organism, with less strength in the abdominal walls, and in every way less tissue, than was possessed by Eng. You can mark that distinction very plainly in the two halves of the band, proving, if we had no other means of proof, that there could not be any very intimate communication of the vessels between the two.

The first point worthy of notice is that of an isolated mass of adipose tissue, evidently sub-peritoneal, which is in the position of the usual umbilicus; namely, in the median line of the abdomen, about half-way up the anterior wall. This is strictly symmetrical, a similar point of about the same size being found in Chang.

Another fact equally well pronounced is that in Chang the bladder was found very much contracted and contained no urine; it was deep down in the cavity of the true pelvis. That of Eng, however, was distended with urine; hence there was a contrast in the appearance of the umbilical fold in the two individuals, in consequence of the great difference in the actual size of the bladder.

My finger is now in the *umbilical pouch* of Chang. The motion is noticeable in the under surface of the band. On the side of Eng no such motion will be observed. I can very clearly see my finger passing between the two folds of the suspensory ligament. At this point it would perhaps be well to exhibit the drawings which have been made of the views which we have been able to obtain from this very limited incision. On looking up towards the band with the greatest possible stretch of tissue, we see the arrangement of the remains of the hypogastric arteries converging towards the bond of union. In this lower diagram we show you the livers joined by what is supposed to be the *tract of portal continuity*. You will observe the limits are somewhat symmetrical. Here is the liver of Chang, with a foreshortened right lobe.

The remainder of the right lobe is deep within the abdomen, and of course it has not been seen. Here is the fundus of the gall-bladder, and there the suspensory ligament, carrying the remains of the umbilical vein. When the finger is passed from Chang into Eng, it is received between the folds of the suspensory ligament of Eng. In Eng, the parts are essentially the same, although you see more evidence of adipose tissue. Here is a little ligament aiding in the support of the liver, to whose convexity it is attached; it is not seen in Chang at all. You might term it an accessory suspensory ligament. When the finger is introduced behind the pouch, it is observed to terminate blindly, showing, we think, that it is adventitious, due to the presence of that suspensory ligament.

We find some vessels of the portal system, even as far down as the mesentery, well filled with the blue coloring-matter. We of course desired, as far as possible, to examine all the tissues here by these incisions; hence it was that when the bodies were in this position the skin was taken off from the wall in order to get a view of the linea alba.

[The bodies were here inspected by the audience, and afterwards turned so as to expose the posterior part of the band. Further remarks apply to this posterior aspect.]

Dr. Pancoast: While the bodies are being turned, I will take the opportunity of replying to one or two questions which have been asked me. First, in regard to the common sensibility of these individuals. According to the statements received at Mount Airy, there was a line of common sensibility corresponding to the median line of the band. Dr. Hollingsworth says that if a pin were stuck into the band at the median line, both of the twins would feel it distinctly; but that, even at a slight distance to either side, the point of the pin produced an effect only on the twin of that side.

Another question has been asked me, as to whether either of them was ever put separately under the influence of an anæsthetic. I answer it by saying that so far we know it never was attempted, but that when, upon the final occasion, Chang was anæsthetized by death, Eng was for a time unaffected. The story as told us at Mount Airy was that Eng waked up and asked his son, "How is your uncle Chang?" The boy said, "Uncle Chang is cold—Uncle Chang is dead." Then great excitement took place. Eng commenced crying out immediately—saying to his wife, whom they called in, "My last hour is come," and finally sank away. He was in perfect health when they went to bed.

They had been sitting up in a large double chair, made for their accommodation. Eng was smoking his pipe, until he became sleepy, and finally said to Chang, "We must retire." Chang said that he could not lie down comfortably. I understand that when they went from Chang's house to Eng's house [see editorial], where they died, it was against the direction of Dr. Hollingsworth; but with their usual stubborness they persisted in riding the distance in an open buggy. To return to the narrative of the night of their death, after Chang had refused to lie down, they walked about the house for some time, and even went out to the porch, and washed their hands and drank some water. It was about 1 o'clock when they went to bed. Then Chang died, some time between that and morning; his death not producing any immediate impression on Eng. It was only when the latter woke up and inquired about the condition of his brother, that he was at all affected.

As to the question, "What caused Eng's death?" I am not able to tell. The post-mortem which has been made does not show the condition of his lungs. Probably the valves of his heart were in a disorganized condition, and probably also the shock upon that weakened organ caused death.

Dr. Allen: In my opinion, Chang died of a cerebral clot. From inquiry at his home, I was led to believe that the lung-symptoms were not due to pneumonia; indeed, were not severe enough to have been so caused. The suddenness of the death, the general atheroma of the arteries, and the fact that there had been previously an attack of cerebral paralysis, all indicated that the death was of cerebral origin. Eng probably died of fright, as the distended bladder seemed to point to a profound emotional disturbance of the nervous system, the mind remaining clear until stupor came on—a stupor which was probably syncopal. One thing to be settled in the making of our examination was to get the bodies in the best possible position, so that we could judge of the true nature of the band.

You will observe the great contrast between the anterior appearance of the band and its posterior aspect. When we suspended them face to face we conceived we had them in the proper position for study. On the posterior side there was a fold underneath the skin extending from the ensiform cartilage of Chang, passing over, crossing the median line, and inserted into the ensiform cartilage of the opposite twin, Eng. It was one of the objects of the examination to determine what was the nature of this fold. I judge it to be the linea alba; but I leave the Fellows to decide that for themselves. I will also add that, because we had not the privilege of cutting the anterior portion of the band, we were obliged to cut down from the point of which I have spoken, to get to the structure, and demonstrate these *culs-de-sac* from behind.

Here, (referring to the casts), from this point the incision is horizontal about midway, and joined laterally by two oblique lines which were directed one upward and the other downward and outward, making a modified letter-H incision. Thus we got all the space we needed. When I raise the skin, we see the scar of the umbilicus in the superficial fascia; and on lifting the other flap we get a better general demonstration.

And now we come upon the point of interest, namely, the position of the band and its true nature. We have a diagram here. You notice on Chang's side that there is an arrangement of interlacing aponeurotic fibres, marked here; and these fibres. starting in Chang, pass across the median line and are inserted into the ensiform cartilage of Eng. Turning the lower flap downward, the upper flap upward, and the two lateral tongues outward, the superficial fascia is exposed. This is abundantly supplied with adipose tissue on either side, but is free from fat where it covered the band. Both the lower flap and the fascia are lost in the scar marking the position of the umbilicus. The same dissection exhibits the position of the lower pouch of Chang. Turning down the external oblique, the two recti, and the internal oblique muscles, the transversalis was exposed, the latter forming a very well-defined layer in Eng, with an interval between the ensiform cartilage and the inferior margin of the thorax. These were much less marked in Chang.

Turning forward this layer of fibres in Eng from without inward, the diaphragm is brought into view. Muscular fibres are conspicuous in this position. The peritoneum on either side is now fairly exposed. Incisions may now be made with a view of demonstrating the pouches of the band. The upper pouch of Chang is, you will observe, freely opened on its posterior aspect, and the vessels in the tract of portal continuity are seen to be well distended with the injecting fluid. A small artery is seen crossing beneath this tract of veins, and is probably a branch of the hepatic; but, whatever may be its origin, it evidently could have little effect in influencing the nutrition of parts beyond the band, and is probably retained within the band itself. The lower pouch of Chang reveals nothing which was not demonstrable from in front, and the same may be said of the single pouch of Eng, thus confirming our opinions of the construction of the band before the pouches had been opened from behind.

Dr. Abraham Jacobi, of New York, being called upon, said: I am very much obliged to the gentleman who has mentioned my name. I do not believe, Mr. Chairman, that I have anything to add to the stock of knowledge in regard to the subject before us. If I were to answer the question as to how this monstrosity originated—especially whether they became connected after having been separate organisms, I should say that that idea has been given up by those whose opinions are entitled to weight. It is true that years ago such specimens were spoken of by Dalton in Holland; and a number of others have alluded to the idea that two such individuals might in embryonic life become united simply by adhesion, the result of their being located together in the embryo. In truth, it appears to me that at that period such a thing might be possible; but of course the union would be a superficial one, not involving the deep organs.

We know that the first epidermis is formed about the end of the fifth week of embryonic life, and that after a time it is thrown off, so that the embryo of about seven or eight weeks is more loosely covered with the real epidermis than in the earlier period. The epidermis is thrown off a number of times until about the fourth month of utero-gestation, when it is finally perfected and remains intact. Now it is suggested that at those times when the epidermis is thrown off the connection takes place between the two individuals—just as the connection takes place between the prepuce and glans, which we so often find adherent in the fœtus and in a number of new-born children.

There are evidences, which we cannot forget, that such connections have taken place before the final epidermis is formed, and about the time one of the earlier coverings is being thrown off, at a period when the internal organs, frequently implicated in such monstrosities, are already formed. There are few double monstrosities so well developed as this one. I think the records of about four hundred monsters have now been collected in the books and journals; but very few are of such a complete nature as this. Every one has heard of the Hungarian Twins, who lived to the age of twenty-one years, in the last century. Another pair of female twins, that traveled in Germany about two years ago, were described at the time, in the *Berliner Wochenschrift.* They were of a similar nature. There are two cases on record in which a division has been successfully attempted, but in those cases the connections were not so well developed as in the Siamese Twins. The connection was in the same neighborhood, but was only superficial—of skin and subcutaneous tissue. One of the cases is recorded by Dr. Braun (*Virchow's Archiv*). Fortunately, or unfortunately, I do not know which, they were his own children. They were of the female sex. He separated them immediately after birth. One lived three and a half days; and when the case was described, in 1866, the other was five years old. In that instance the connection—three and a half inches long—extended from the ensiform process to the umbilicus. The other case is described as early as 1689, by the old German author, Kernoch.

As far as the origin of twin monsters is concerned, I am certainly of those who are not of the opinion that two individuals could get into such an intimate connection by growing together. Certainly the connection is an original one. I believe that the general opinion is now that one Graafian vesicle may have two ova, or one ovum have two nuclei; and these finally may, like the two vitelli of an egg, be closed together, surrounded by the same material, forming a single complete ovum; and thus it may be that the two are included in the same ovum. I think that this will explain also why the sex is always the same—why they are always both male or both female. They are male in twenty or twenty-five per cent. of the cases.

Dr. H. C. Wood here asked Dr. Jacobi a question in regard to the Biddenden Sisters (an account of whom will be found in another column of this journal), as to whether they had been reported in the works on monstrosities.

Dr. Jacobi: I do not know anything about that.

Dr. Pancoast stated that an account of those sisters was contained in a semi-popular book entitled "Lexicon Tetraglotton," published by Samuel Thomson, London, 1660.

None of the Fellows desiring to say anything further upon the subject, on motion, the college adjourned.

